# Polymorphisms Contributing to Calcium Status: A Systematic Review

**DOI:** 10.3390/nu13082488

**Published:** 2021-07-21

**Authors:** Katharina da Silva Lopes, Sarah Krull Abe

**Affiliations:** 1 Graduate School of Public Health, St. Luke’s International University, Tokyo 104-0045, Japan; 2 Center for Public Health Sciences, National Cancer Center, Division of Prevention, Tokyo 104-0045, Japan; saabe@ncc.go.jp

**Keywords:** calcium requirements, calcium homeostasis, SNPs, polymorphism, genetic factors

## Abstract

This systematic review assessed genotypes and changes in calcium homeostasis. A literature search was performed in EMBASE, Medline and CENTRAL on 7 August 2020 identifying 1012 references. Studies were included with any human population related to the topic of interest, and genetic variations in genes related to calcium metabolism were considered. Two reviewers independently screened references, extracted relevant data and assessed study quality using the Q-Genie tool. Forty-one studies investigating Single Nucleotide Polymorphisms (SNPs) in relation to calcium status were identified. Almost half of the included studies were of good study quality according to the Q-Genie tool. Seventeen studies were cross-sectional, 14 case-control, seven association and three were Mendelian randomization studies. Included studies were conducted in over 18 countries. Participants were mainly adults, while six studies included children and adolescents. Ethnicity was described in 31 studies and half of these included Caucasian participants. Twenty-six independent studies examined the association between calcium and polymorphism in the calcium-sensing receptor (*CASR*) gene. Five studies assessed the association between polymorphisms of the Vitamin D receptor (*VDR*) gene and changes in calcium levels or renal excretion. The remaining ten studies investigated calcium homeostasis and other gene polymorphisms such as the *CYP24A1* SNP or *CLDN14*. This study identified several *CASR*, *VDR* and other gene SNPs associated with calcium status. However, to provide evidence to guide dietary recommendations, further research is needed to explore the association between common polymorphisms and calcium requirements.

## 1. Introduction

As one of the most important minerals in the human body, calcium fulfils essential physiological roles such as mineralization of bones and teeth, muscle contraction, blood clotting and transmission of nerve impulses [1]. Ninety-nine percent of total calcium in the body is in bones and teeth [1]. The remaining one percent can be found in blood and body fluids [2]. Its homeostasis is tightly regulated and intra- and extracellular concentration of calcium are maintained through the activation and deactivation of signalling cascades involving mainly the hormone systems parathyroid hormone (PTH) and vitamin D [3,4].

The recommended dietary allowances (RDA) for calcium by the American Institute of Medicine (IOM) is 1000 mg/day for adults aged 19–50 and men until age 70 and 1200 mg/day for women >50 years and men >70 years [5]. The IOM identified the revised tolerable upper intake level for calcium as 2500–3000 mg/day for adults [5]. These levels were determined considering the following indicators: hypercalcemia, hypercalciuria, vascular and soft tissue calcification and nephrolithiasis [5]. Calcium balance changes during the life stages and is determined by dietary calcium intake, intestinal calcium absorption and renal reabsorption [1].

Inadequate intake of calcium or increased calcium requirements can lead to calcium deficiency. Long-term calcium deficiency, disruption of PTH secretion or the vitamin D metabolism can cause chronic low levels of serum calcium (hypocalcemia) [6]. Symptoms of low calcium levels may include muscle aches, fatigue, skin sensitivity and in more extreme cases bone fractures due to demineralization, convulsions, arrhythmias and death [6]. The major causes of hypercalcemia or too much calcium are increased bone resorption due to high levels of PTH or increased intestinal calcium absorption due to high levels of vitamin D [7]. Increased levels of serum calcium can weaken bones, lead to the formation of kidney stones, and intervene with functions of the heart and brain [7]. Calcium status depends on multiple factors including genetics. Single nucleotide polymorphisms (SNPs) of the calcium-sensing receptor (CASR) such as SNPs rs1801725 A986S [8,9], rs1042636 R990G [10] or rs1801726 Q1011E [11] as well as the vitamin D receptor (VDR) SNP *Bsm*1 (rs1544410) [12] and *Fok*1 (rs2228570) [13] have been suggested to be associated with calcium status. 

Normal levels of total serum calcium range between 2.12 and 2.62 mmol/L (8.5 and 10.5 mg/dL) [1]. The CASR responds to changes in extracellular calcium. Low levels of extracellular calcium result in signals from the CASR to increase PTH secretion in the parathyroid gland and to decrease thyroid calcitonin (opposing hormone to PTH) secretion [14,15]. To elevate calcium levels to normal ranges, PTH stimulates the mobilization of calcium from bones, absorption of calcium in the intestine and reabsorption in the kidney through increased activation of vitamin D [15,16]. In a feedback mechanism, the increase in calcium results in the suppression of PTH secretion and stimulation of the release of calcitonin through signals from the CASR [1,15]. Calcitonin reduces the uptake of calcium in the kidney and stimulates calcium deposition in the bones [15]. Genetic variations of the *CASR* gene can lead to disturbances in calcium homeostasis through gain-of-function mutations resulting in a receptor with higher activity, e.g., *CASR* SNP R990G [17] or a less active receptor as seen for the *CASR* SNPs A986S [18] and Q1101E [19].

It is not known if polymorphisms of genes involved in calcium regulation result in different dietary requirements of calcium depending on the genotype. Therefore, we systematically assessed genetic factors and calcium status in this review and aimed to summarize the effects of different polymorphisms on calcium homeostasis. 

The review was commissioned to inform the work of updating the joint Food and Agriculture Organization of the United Nations (FAO, Rome, Italy) and World Health Organization (WHO, Geneva, Switzerland) calcium requirements in children 0–36 months of age.

## 2. Materials and Methods

We followed the Preferred Reporting Items for Systematic Reviews and Meta-Analyses (PRISMA) guideline [20]. The review was registered at the International Prospective Register of Systematic Reviews (PROSPERO) under CRD42021240324.

### 2.1. Search Methods

**Figure 1:**
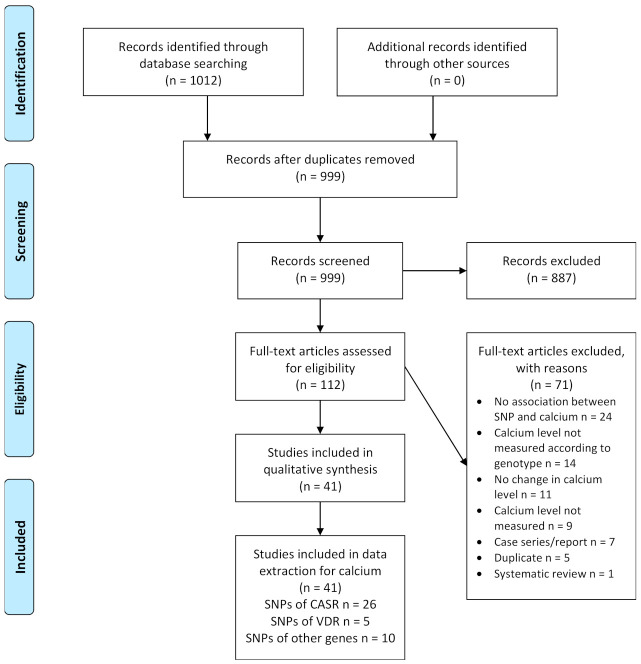
Study flow diagram. Abbreviations: CASR: calcium-sensing receptor; SNP: single nucleotide polymorphism; VDR: vitamin D receptor.

We conducted a literature search in the following databases from inception to 7 August 2020: CENTRAL, EMBASE and Medline (Figure 1). We did not apply any restrictions regarding publication type, date or language. A detailed search strategy is included in the supplement (Supplementary Table S1). 

### 2.2. Inclusion Criteria

Human studies were included related to the topic of interest and genetic variations in genes linked to calcium metabolism were considered. We included any population regardless of age, gender or health status. For context, changes in metabolic status of calcium (e.g., serum calcium concentration, ionized calcium concentration and urinary calcium excretion, calcium absorption) were assessed in any way as no robust biomarker is currently available. Observational and intervention studies were included. Additionally, abstracts were included if the full study was not published. Reviews and systematic reviews were used to identify primary studies for inclusion. 

### 2.3. Exclusion Criteria

Studies reporting results on gene variants unrelated to calcium metabolism were excluded. Studies which did not mention the metabolic status of calcium (e.g., serum calcium concentration, ionized calcium concentration, urinary calcium excretion, calcium absorption) were excluded. Studies only describing clinical outcomes e.g., higher/lower disease risks without describing changes in calcium, were also excluded. Case reports were excluded as these might describe only rare genetic variants. Other study designs which were excluded were animal and in vitro studies. Abstracts were excluded if the study was already published as a full article.

### 2.4. Data Collection and Analysis

#### 2.4.1. Selection of Studies

The two reviewers independently screened the titles and abstracts of the studies using Rayyan [21]. We retrieved full-text articles and assessed them for inclusion. We resolved conflicts through discussion. We manually searched reference lists and reviews of relevant studies.

#### 2.4.2. Data Extraction

We developed an extraction table including the following information: basic study characteristics (year of publication, country, study type), sample description (total number of participants, male and females, age, ethnicity, inclusion and exclusion criteria) and association of genetic variants with calcium status. The two review authors extracted the data into a table which included: SNPs associated with the phenotype (gene, rs#, nucleotide change, amino acid change), prevalence of SNP in the study population, description of SNP and phenotype. SNP association with calcium change including SNP association with calcium intake or calcium supplementation was also included if available. To assess changes in calcium status, the following outcomes were extracted: serum calcium concentration, ionized calcium concentration, urinary calcium excretion or calcium absorption. The data were cross-verified. Any inconsistencies were resolved through discussion.

### 2.5. Assessment of Study Quality

We used the Quality of Genetic Studies (Q-Genie) tool version 1.1 [22]. The Q-Genie tool contains 11 questions and a quality assessment marked on a 7-point Likert scale ranging from 1 (poor) to 7 (excellent).

The method has been previously described in detail and the assessment tool is available [22]. Briefly, one author respectively assessed all studies, while the other author confirmed assessment. Disagreements were settled through discussion. We assessed the included studies covering the following themes: scientific rational, ascertainment of comparison groups, technical and non-technical classification, outcome (e.g., disease status or quantitative trait), bias, sample size, planned statistical analyses, statistical methods, test of assumptions and interpretation of results [22]. For studies with control groups, total scores of ≤35 indicate poor quality studies, >35 and ≤45 indicate studies of moderate quality, and >45 indicate good quality studies. For studies without control groups, total scores of ≤32 indicate poor quality studies, >32 and ≤40 indicate studies of moderate quality and >40 indicate good quality studies.

## 3. Results

### 3.1. Results of the Search

The literature search in three databases identified 1012 references. After removal of duplicates, a total of 999 title and abstracts were independently screened by the two review authors. We excluded 887 records and the two review authors independently assessed 112 full-text articles for inclusion by applying the pre-specified eligibility criteria. Of the 71 records excluded at this stage, 24 articles did not show an association between the studied polymorphism and calcium, in 14 articles the calcium levels were not measured according to genotype, no changes in calcium levels were seen in 11 articles, nine did not measure calcium levels at all, seven were case series or case reports, five were duplicates and one article was a systematic review. Finally, we included 41 studies for qualitative synthesis. The process of study selection is shown in the PRISMA flow diagram (Figure 1).

### 3.2. Assessment of Study Quality

We assessed the quality of included studies using the Q-Genie tool (Table 1). Nine-teen studies (46%) were of good quality [9,12,19,23,24,25,26,27,28,29,30,31,32,33,34,35,36,37,38], 16 studies (39%) of moderate quality [8,11,13,39,40,41,42,43,44,45,46,47,48,49,50,51] and six studies (15%) of poor quality [10,52,53,54,55,56]. Most studies sufficiently provided a rational for the selected gene or genes in the study and appropriately drew conclusions supported by the presented results. Studies only available as abstracts [8,10,39,53] provided very few methodological details and many items were not sufficiently clear to make a judgement about the study quality. Prior power analysis and an appropriate sample size were not provided in most of the studies. Furthermore, non-technical classification of the exposure was problematic in almost all studies due to insufficient information about the blinding of the assessor who conducted the genotyping or details about the randomisation of samples prior to genotyping.

### 3.3. Description of Included Studies

Characteristics of the included 41 studies are summarized in Table 2, Table 3, Table 4. Studies were carried out between 1999 [13,40,49] and 2019 [9,35]. Twenty-one studies were conducted in Europe [8,10,11,26,27,28,29,30,31,32,33,38,39,42,43,44,49,50,51,52,54], 10 in Asia [12,24,36,37,45,47,48,53,55,56] and six in North America [13,19,23,25,40,41]. Three studies [9,34,35] included cohorts from different countries while one study provided no country information [46]. Of the included studies, 17 were cross-sectional studies [8,11,13,27,30,33,34,37,38,39,41,45,49,50,51,53,56], 14 were case-control studies [10,12,19,24,29,31,32,36,42,43,47,48,52,55], seven association studies [23,25,26,35,40,46,54] and three were Mendelian randomisation studies [9,28,44]. Twenty-six studies examined the association between calcium and polymorphisms in the *CASR* gene (Table 2). Five studies examined polymorphisms in the *VDR* gene and calcium homeostasis (Table 3). Ten studies investigated other gene polymorphisms including *AHSG* (alpha 2-HS glycoprotein), *CALCR* (calcitonin receptor), *CLDN14* (claudin-14), *CYP24A1* (cytochrome P450 family 24 subfamily A member 1), *DGKD* (diacylglycerol kinase delta), *GCKR* (glucokinase regulatory protein), *GNAS1* (guanine nucleotide binding protein alpha subunit), *hKLK1* (human renal kallikrein), *LPH* (lactase-phlorizin hydrolase); *NMU* (neuromedin U) and *ORAI1* (calcium release-activated calcium modulator 1) (Table 4). 

### 3.4. Description of Participants in Included Studies

The total number of participants in included studies ranged from 72 [13] to 184,205 [28]. Six studies included only female participants [19,40,41,44,45,53] and two only male participants [47,49]. Five studies were conducted in children [13,23,34,52,54], one in adolescent girls [44] and in one study it was unclear if patients included only adults or also children [10]. The remaining studies included adults of different ages. Ethnicity was described in 31 studies: 15 studies included Caucasians [11,27,29,32,34,38,39,40,41,42,44,49,50,51,54], two studies Indians [24,55], Japanese [12,56], one study Africa-Americans [25], Chinese [53], Korean [37], Taiwanese [36] and eight studies included multi-ethnic participants [9,13,19,23,26,28,35,46]. In 10 studies, no information on participants’ ethnicity was provided [8,10,30,31,33,43,45,47,48,52]. Twenty-two studies included healthy participants [8,9,11,13,23,25,26,27,34,37,38,39,40,41,43,44,46,49,51,53,54,56], six studies included patients with kidney or urinary stones [24,32,35,47,48,55], four with primary hyperparathyroidism (PHPT) [10,30,31,42], two with coronary artery disease (CAD) [28,29] or hypertension [12,33], and one with vitamin D deficiency (VDD) [45], hypercalciuria [52], cancer [19], cardiovascular risk factors [50] or chronic kidney disease (CKD) [36]. 

### 3.5. Calcium and Polymorphism of the Calcium-Sensing Receptor (CASR)

Twenty-six independent studies were identified that assessed the association of polymorphisms of the *CASR* gene and changes in calcium levels or renal excretion (Table 2). Seventeen out of 26 studies examined the SNP rs1801725 (G/T, A986S, Ala > Ser) [8,9,11,19,23,24,26,27,28,29,32,40,41,44,45,48,52]. Except three studies which looked at renal calcium excretion [32,48,52], all studies assessed the association between SNP and serum calcium levels. Healthy women homozygous for A986 (GG) had lower serum calcium levels corrected for albumin compared with heterozygotes A986S (GT) [40] and the result was confirmed for serum calcium levels without correction for albumin in a follow-up study with a greater number of participants [41]. Ionized calcium levels were lower in healthy adults with the common GG genotype compared with GT or TT genotypes [11,27]. Kidney stone formers [24] or breast cancer patients [19] as well as healthy controls with the more common genotype GG showed lower serum calcium levels compared with GT and TT genotypes together. Cerani et al. investigated various SNPs in different genes and found an increase in serum calcium by 0.0178 mmol/L (0.0713 mg/dL) for the calcium increasing allele T in *CASR* rs1801725 [9]. Also, Larsson et al. showed an increase in serum calcium by 0.0177 mmol/L (0.071 mg/dL) per additional T allele in patients with CAD [28] and each T allele yield a calcium increase of 0.01874 mmol/L (0.0751 mg/dL) in a large genome-wide association study [26]. März et al. reported significantly higher serum calcium levels and calcium levels adjusted for albumin for patients with or without CAD carrying the GT or TT genotype compared with the GG genotype [29]. Healthy carriers of the T allele had significantly higher serum calcium levels [8] or higher serum calcium levels corrected for albumin in another study of healthy females [44]. One study also showed an association of the SNP with serum calcium level for the minor allele T in African-Americans and European-Americans [23]. No association between different genotypes and serum calcium level was found in adult women with VDD [45]. In kidney stone formers, carriers of the different alleles for the SNP rs1801725 reported no differences in 24 h urinary calcium excretion or serum calcium concentration between wild type and mutant [48]. In one study examining the three common *CASR* SNPs rs1801725 (A986S), rs1042636 (R990G) and rs1801726 (Q1011E) in kidney stone formers and healthy controls, higher urinary calcium excretion was reported in participants with rs1801725 GG, rs1042636 GG or AG, rs1801726 CC genotype compared with participants with rs1801725 GG, rs1042636 AA, rs1801726 CC genotype [32]. One study investigated the SNP rs1801725 in children with idiopathic hypercalciuria and healthy controls and found a positive association in the T allele with renal calcium excretion, independent of age and serum levels of calcium, intact parathormone and 25-hydroxy-vitamin D [52].

The second most studied polymorphism of the *CASR* gene was rs1042636 (A/G, R990G, Arg > Gly) which was investigated in 11 studies [8,10,11,24,30,32,42,45,48,52,53]. The common AA genotype showed higher ionized calcium levels compared with the AG genotype in healthy adults [11] and also higher serum calcium levels for the AA genotype compared with the genotypes AG or GG in adult females with VDD [45], healthy young Chinese women [53], or kidney stone formers and healthy controls [48]. Furthermore, in one study examining the association of the three most common SNPs rs1801725, rs1042636 and rs1801726 and calcium, carriers of TG (rs1801725) AA (rs1042636) GC (rs1801726) had significantly higher serum calcium levels compared to the most common haplotype GG AA CC [8]. In contrast, PHPT patients carrying minor alleles in two *CASR* SNPs rs1042636 (GG or GA) and rs1501899 (AA or GA) had higher serum ionized calcium and higher urine calcium compared with patients carrying wild type alleles at both SNPs [30]. PHPT patients [10,42] or kidney stone formers [24,32] carrying the GG or AG genotype showed higher urinary calcium excretion compared with homozygotes for the 990R allele (AA genotype). One study investigated the SNP rs1042636 in children with idiopathic hypercalciuria and healthy controls, but did not report changes in calcium levels or renal excretion [52].

Five studies assessed the *CASR* SNP rs1801726 (G/A,C, Q1011E, Gln > Glu) [8,11,32,48,52]. Of those, three showed higher serum calcium levels for the GC genotype compared with the more common CC genotype in healthy participants [8,11] as well as kidney stone formers [48]. In kidney stone formers and healthy controls, heterozygous participants with GC had lower urinary calcium excretion compared with CC homozygotes, but there were no differences in plasma calcium levels [32]. One study investigated the SNP rs1801726 in children with idiopathic hypercalciuria and healthy controls, but did not report on changes in calcium levels or renal excretion [52].

Two studies examined the SNP rs17251221 (A/G) [43,46]; Jorde et al. showed significantly higher serum calcium levels for the GG genotype compared with the AA genotype [43]. O’Seaghdha et al. also confirmed an association of the SNP with 0.015 mmol/L (0.06 mg/dL) higher serum calcium levels per copy of the minor G allele in healthy participants [46].

One study identified an association between *CASR* rs11716910 (A/G) and serum calcium levels (corrected for albumin) and showed that healthy individuals with the AA genotype had higher levels than those with the AG and GG genotypes [39]. Jung et al. investigated 15 SNPs in the *CASR* gene and found that the six SNPs rs6438712 (A/G), rs4678172 (G/T), rs9874845 (A/T), rs4678059 (A/T), rs1965357 (C/T) and rs937626 (A/G) were associated with lower urinary calcium excretion for African-Americans carrying the minor alleles [25]. Vezzoli et al. 2011 looked at two SNPs in the regulatory region of the *CASR* gene in PHPT patients and healthy controls: rs7652589 (G/A) and rs1501899 (G/A) [31]. They found higher serum ionized calcium and higher urine calcium for PHPT patients with the diplotype AA/AA or AA/GG versus GG/GG [31].

### 3.6. Calcium and Polymorphism of the Vitamin D Receptor (VDR)

Five studies [12,13,49,54,55] assessed the association between polymorphisms for the *VDR* gene and changes in calcium levels or renal excretion (Table 3). Three studies examined the *VDR* SNP *Bsm*1 (rs1544410, A/G) [12,49,55]. In healthy subjects, intake of a high calcium-phosphate diet resulted in higher calcium concentrations in fasting urine and higher daily calcium excretion in subjects carrying the mutant bb genotype compared with the wild type BB genotype, but there was no difference between genotypes for serum ionized calcium, urinary calcium or daily urinary calcium excretion without dietary modifications [49]. Participants consumed products rich in calcium and phosphate and additionally 1000 mg elemental phosphorus per day as potassium-phosphorus syrup for five days (high calcium-phosphate diet) [49]. Urinary calcium excretion was higher for the bb genotype in nephrolithiatic subjects with or without hypercalciuria [55] and total and ionized serum calcium levels were lower in normotensive or hypertensive subjects with the BB genotype [12]. 

Two studies examined the *VDR* SNP *Fok*1 (rs2228570, C/T) [13,55]. Healthy children with the wild type FF genotype showed a greater calcium absorption compared with the mutant ff or Ff [13]. Serum calcium was significantly higher in hypercalciuric nephrolithiatic subjects with the ff genotype compared with FF or Ff genotypes and FF and Ff genotypes showed higher renal calcium excretion compared with ff [55]. 

In another study, the *VDR* SNP rs4516035 (−1012 G/A) was significantly associated with serum calcium levels (adjusted for serum protein levels) and the GG genotype in healthy children and adolescents showed lower levels compared with GA or AA genotypes [54].

### 3.7. Calcium and Polymorphism of Other Genes

Ten independent studies examined the association between calcium homeostasis and polymorphisms in various genes (Table 4). Seven studies [34,35,36,37,38,50,51] found a link with serum calcium levels and three with renal calcium excretion [33,47,56]. In patients with one or more cardiovascular risk factors, the *AHSG* (alpha 2-HS glycoprotein) SNP rs4918 (G/C, T256S) was associated with lower calcium levels for patients carrying the CC genotype compared with the GC or GG genotypes [50]. Another study by Gianfagna et al. examined *NMU* (neuromedin U) SNP rs9999653 (major/minor C/T) and found a significant association between the CC genotype and lower serum calcium levels [34]. In a multi-ethnic study on kidney stone disease, *CYP24A1* (cytochrome P450 family 24 subfamily A member 1) SNP rs17216707 (T/C) showed higher serum calcium levels for participants carrying the TT genotype compared with those carrying the TC but not CC genotype [35]. In the same study, *DGKD* (diacylglycerol kinase delta) rs838717(A/G) was associated with lower 24 h renal calcium excretion for male patients carrying the AA genotype compared with GG [35]. Hwang et al. looked at the influence of the SNP rs12313273 (C/T) in the *ORAI1* (calcium release-activated calcium modulator 1) gene in CKD patients and found that serum calcium concentration was significantly higher in carriers of the C allele compared with T carriers [36]. One study examined the association of the SNPs rs780093 (T/C), rs780094 (T/C) and rs1260326 (T/C) in the *GCKR* (glucokinase regulatory protein) gene in healthy participants [37]. Participants with the minor CC genotype displayed significantly decreased serum calcium levels compared with participants with the TT genotype for all three investigated SNPs. One study identified an association between the *LPH* (lactase-phlorizin hydrolase) polymorphism rs498823 (G/C) and calcium levels, where C-homozygotes had lower ionized calcium levels compared with G homo- or heterozygotes, but there was no difference in total serum calcium levels between genotypes [38]. Another study assessed the *GNAS1* (guanine nucleotide binding protein alpha subunit) gene polymorphism (T/C) and showed that healthy participants with the minor C allele had lower serum calcium levels compared with the major T allele [51].

One study looked at the different SNPs in the 3′ region of the claudin-14 gene and found the strongest association between the *CLDN14* SNP rs219755 (G/A) and 24 h-urinary calcium excretion with higher excretion for the GG genotype compared with GA or AA in patients with hypertension [33]. In healthy Japanese volunteers, the polymorphism in the *hKLK1* (human renal kallikrein) gene (promoter region, H allele: with nucleotide substitution 130(G)11) was associated with higher fractional calcium excretion in the urine and higher calcium excretion per creatinine in the urine of subjects with the H allele compared with subjects without the H allele [56]. Shakhssalim et al. 2014 studied different SNPs in the *CALCR* (calcitonin receptor) gene and found significantly higher urine calcium concentrations for hetero- or homozygotes (patients with T allele) compared with the wild type for the 3′UTR + 18C > T polymorphism in kidney stone formers (calcium urinary stones) [47].

## 4. Discussion

Calcium is an essential micronutrient critical to human physiology, particularly bone and dental health. It cannot be produced in the body and needs to be obtained through a balanced diet. Genetic factors, such as polymorphisms of genes involved in calcium signalling, can influence calcium homeostasis [26,36]. However, it is unknown whether these genetic variations directly affect dietary requirements of calcium. In this context, our systematic review aimed to assess and summarize genetic factors contributing to changes in calcium status. We identified 41 studies for inclusion and about half (46%) were of good study quality. Twenty-six studies examined the association between polymorphism in the *CASR* gene and changes in serum calcium levels or renal calcium excretion, five between polymorphism in the *VDR* gene and calcium and 10 studies investigated the relationship between different gene polymorphisms and calcium.

The extracellular CASR plays a pivotal role in maintaining calcium homeostasis and is highly expressed in the parathyroid glands and the kidneys [14]. To regulate calcium levels, the receptor detects changes in extracellular calcium concentration and mediates the secretion of PTH and calcium absorption in the kidney [14,57]. Various mutations in the *CASR* gene leading to diseases such as familial hypocalciuric hypercalcemia (FHH; loss-of-function mutation) or hypocalcemia (gain-of-function mutation) have been described [46]. Polymorphisms of the *CASR* gene have been associated with changes in calcium status; the three polymorphisms A986S (rs1801725), R990G (rs1042636) and Q1011E (rs1801726) have been intensely studied and seem to be of clinical importance [8,11,32,48,52]. The polymorphism A986S (rs1801725) results in a shift from alanine to serine at codon 986 and probably in a less active receptor [18]. Participants carrying the minor T allele had significantly higher serum calcium levels [8,9,11,19,23,24,26,27,28,29,40,41,44]. This observation was independent of the health status of study participants leading to the speculation that healthy individuals [8,9,11,23,26,27,40,41,44], kidney stone formers [24], breast cancer patients [19] and CAD patients [28,29] with the minor genotypes GT or TT may have lower calcium requirements compared with individuals carrying the major genotype GG. However, none of these studies examined dietary calcium intake or calcium supplementation.

R990G (rs1042636) and Q1011E (rs1801726) are also common polymorphisms of the *CASR* gene and have been associated with calcium levels [11]. The *CASR* polymorphism rs1042636 leads to an amino acid change from arginine to glycine at codon 990 which results in a receptor with higher activity [17]. The common AA genotype showed higher calcium levels compared with the mutant AG or GG genotype in healthy study participants [11,53], women with VDD [45] or kidney stone formers [48]. Furthermore, PHPT patients [10,42] or kidney stone formers [24,30,32] carrying the mutant AG or GG genotype had higher urinary calcium excretion compared with the wild type AA genotype. The *CASR* SNP rs1801726 (Q1011E) results in a cytosine-guanine substitution at codon 1011 and reduced receptor function [19]. The mutant GC genotype led to higher serum calcium levels compared with the wild type (CC) in healthy individuals [8,11] as well as kidney stone formers [48]. Our review also identified studies examining less common SNPs in the *CASR* gene which resulted in alteration of serum calcium levels and/or renal calcium excretion [25,31,39,43,46]. Depending on the genotype expressed, these studies suggest an association between the polymorphism and calcium homeostasis which potentially could influence calcium requirements. Dietary recommendations for calcium according to the expressed genotype need to take into account the interaction of different polymorphisms and calcium homeostasis as well as calcium intake. The study of the interaction between dietary factors and common polymorphism can help us understand calcium requirements for good health and in disease [58,59]. For example, previous reviews have investigated genetic variants of zinc-transporters and dietary zinc requirements [60], genetic factors influencing vitamin D deficiency [61] or the effects of polymorphism in folate and vitamin B12 genes on metabolism and intake [62]. The gene-nutrient interactions are complex and interpretation of results can be challenging; nevertheless, the studies identified in this review clearly demonstrate changes in calcium status depending on the genotype expressed. These polymorphisms of the *CASR* gene may be interesting candidates to study the gene-nutrient interaction and an individual’s response to differences in calcium intake or supplementation. However, included studies failed to report important confounders such as calcium intake of participants according to genotype. This makes it difficult to compare results from different studies and to draw conclusions about SNPs and the regulation of calcium. Therefore, further research should consider potential lifestyle-related confounders when conducting studies and interpreting results. Furthermore, it would be important to investigate if any individuals are at risk of adverse calcium-related outcomes that would require the need for higher or lower calcium intake.

Vitamin D in its active, hormonal form (calcitriol or 1,25-dihydroxyvitamin D3) binds to the nuclear VDR and mediates calcium homeostasis through three mechanisms: increasing calcium absorption in the intestine, stimulating calcium release from bones and reabsorption of calcium in the kidney [1,3]. The latter two processes require PTH [1]. Mutations in the *VDR* gene can hinder the effect of vitamin D on calcium regulation and may result in diseases such as vitamin D-resistant rickets type 2a (VDDR2A), a condition described with hypocalcemia, hyperparathyroidism and rickets [3,63]. Among the five studies included in this review that assessed polymorphisms in the *VDR* gene and calcium changes, three examined the SNP *Bsm*1 (rs1544410, A/G) which is located in an intronic region of the receptor [12,49,55]. In one study, the wild type allele BB was associated with lower serum calcium compared with the mutant genotype bb [12] while the other two studies looked at renal calcium excretion [49,55]. Ferrari et al. was the only study which assessed the influence of dietary modifications and calcium changes according to genotype [49]. Participants in this study consumed a diet restricted in calcium and phosphate for the first five days, achieved through counselling and the intake of magnesium- and aluminium-containing phosphorus binder. After a washout period, participants received a diet with products rich in calcium and phosphate plus 1000 mg elemental phosphorus per day for five days. Phosphorus is another essential mineral of bones and teeth and its homeostasis is regulated by PTH and calcitriol [64]. This study found higher fasting urinary calcium and higher daily calcium excretion in the urine in healthy participants receiving a high or low calcium-phosphate diet with the bb genotype compared with the wild type BB [49]. Furthermore, renal phosphate (salt of the phosphorus oxoacid) reabsorption capacity and plasma levels were higher in participants with the bb genotype compared with BB, an effect independent of dietary modification (high or low calcium-phosphate diet). This study suggests that polymorphisms in the *VDR* gene interact with dietary factors and the response to changes in the diet depends on the genotype expressed. However, different dietary sources of calcium or phosphorus and their influence on the observations were not examined and discussed in the study. Two studies examined the *Fok*1 (rs2228570, C/T) polymorphism in the start codon of the *VDR* gene and found greater calcium absorption for the wild type FF genotype in healthy children [13], but hypercalciuric nephrolithiatic participants with the FF or Ff genotype showed lower serum calcium levels and higher renal calcium excretion [55]. The results suggest that genetic variations in the *VDR* gene result in change of function of the receptor which alters calcium homeostasis. However, the relevance of these SNPs and changes in serum or urinary calcium in response to calcium intake or supplementation need to be further investigated.

In addition to the aforementioned SNPs in *CASR* and *VDR* gene, our review identified a number of polymorphisms in genes which may play a direct or indirect role in calcium homeostasis. For example, the enzyme CYP24A1 is involved in vitamin D metabolism and is responsible for the degradation of calcitriol [1,35]. The SNP rs17216707 (T/C) in the *CYP24A1* gene resulted in higher serum calcium levels for individuals carrying the TT genotype compared with the TC genotype due to a reduced enzyme activity [35]. The authors suggested that individuals with the TT genotype may be more sensitive to vitamin D and supplementation with vitamin D could potentially increase the risk of kidney stones due to the development of hypercalcemia [35].

Several included studies reported on genetic factors influencing renal calcium excretion [10,24,25,30,31,32,33,35,47,48,49,52,55,56]. However, none of the included studies in this review assessed faecal calcium excretion. Previous research has shown that dietary calcium intake has an influence on calcium homeostasis (e.g., calcium absorption, renal and faecal excretion) and other dietary factors such as faecal fat excretion [65,66,67]. Further studies should take these relationships into account and also assess potential influences of polymorphisms on faecal calcium excretion.

During phases of development, sufficient intake of minerals is extremely important for the growing child. Calcium requirements increase for bone mineralization especially during the neonatal period and puberty [1,68]. For example, the favourable FF genotype of the VDR *Fok*1 polymorphism resulted in greater calcium absorption and higher bone mineral density in prepubertal and pubertal children [13]. This is consistent with another study showing 20% less calcium absorption and bone mineralization during pubertal growth for the ff genotype compared with the FF genotype [69]. Polymorphisms in *CASR* or *VDR* mediated regulation of calcium homeostasis can affect bone formation and growth in children and adolescents [13,23,34,44,52,54]. However, studies lack important information such as calcium intake (according to genotype) and physical activity which can have an influence on mineral levels in children and adolescents. 

Polymorphisms in the *CASR*, *VDR* gene or other genes such as *CYP24A1*, *GNAS1* or *DGKD*, may contribute to the genetic regulation of calcium status. However, except one study, none of the included studies explored gene-nutrient interactions in response to calcium intake or supplementation. Further investigations are needed to explore this relationship. This may reveal if individuals carrying a particular genotype respond to dietary factors differently and require individual recommendations for dietary calcium intake or supplementation. 

## 5. Conclusions

This review assessed genetic variations and calcium homeostasis. We identified several studies assessing polymorphisms in the *CASR* and *VDR* gene and other genes directly or indirectly involved in the regulation of calcium status. Studies showed changes in calcium levels or renal excretion according to the SNP and it may be of importance to consider if individuals have different calcium requirements depending on the genotype expressed. However, with the exception of one study, none of the included studies assessed the interaction of genes and nutrients through dietary supplementation studies. Therefore, it is unclear if different genotypes lead to different calcium requirements. This highlights the need for further research to explore the effects of genetic variants of genes involved in calcium homeostasis and calcium intake through the diet or supplementation. The results of this review should be interpreted with caution and further research is necessary to develop recommendations for calcium requirements that consider polymorphisms known to change calcium status.

## Figures and Tables

**Table 1:** Risk of bias assessment using the Q-Genie tool.

Study Reference (Study Design)	1. Rationale for Study	2. Selection and Definition of Outcome of Interest (Cases/Disease Status or a Quantitative Trait)	3. Selection and Comparability of Comparison Groups (if Applicable)	4. Technical Classification of the Exposure	5. Non-Technical Classification of the Exposure	6. Other Sources of Bias	7. Sample Size and Power	8. A priori Planning of Analyses	9. Statistical Methods and Control for Confounding	10. Testing of Assumptions and Inferences for Genetic Analyses	11. Appropriateness of Inferences Drawn from Results	Total Score
CASR												
Annerbo 2015 ^#^ [8] (Cross-sectional)	4	4	0	4	2	1	1	4	2	4	7	33
Arcidiacono 2009 ^#^ [10] (Case-control)	4	4	4	4	2	1	1	4	2	2	7	35
Bochud 2011 ^#^ [39] (Cross-sectional)	6	6	0	4	2	1	1	4	6	3	7	40
Cerani 2019 [9] (Mendalian randomisation study)	6	6	4	4	2	4	4	5	6	5	7	53
Chang 2015 [23] (Genome-wide association study)	6	4	0	6	3	2	6	5	6	3	7	48
Cole 1999 [40] (Cross-sectional)	6	5	0	3	2	2	2	4	5	4	5	38
Cole 2001 [41] (Cross-sectional)	7	5	0	3	2	2	4	3	4	3	5	38
Corebetta 2006 [42] (Case-control)	6	6	6	5	2	2	2	4	5	2	5	45
Guha 2015 [24] (Case-control)	6	5	7	4	2	2	6	5	4	2	6	49
Jorde 2013 [43] (Case-control)	7	6	5	4	2	3	2	4	3	2	6	44
Jung 2009 [25] (Population-and family-based association study)	6	6	0	6	2	2	5	3	4	5	6	45
Kapur 2010 [26] (Meta-analysis of genome-wide association study)	7	4	4	2	3	3	7	6	6	5	6	53
Laaksonen 2009 [27] (Cross-sectional)	6	6	0	4	3	3	2	3	6	4	6	43
Larsson 2017 [28] (Mendalian randomisation study)	6	5	4	4	3	6	6	7	5	5	6	57
Lorentzon 2001 [44] (Association study)	6	4	0	2	2	2	4	4	4	2	5	35
Majid 2015 [45] (Cross-sectional)	7	5	0	4	2	3	2	4	3	4	6	40
März 2007 [29] (Case-control)	6	6	4	3	6	2	4	5	4	2	6	48
O’Seaghdha 2010 [46] (Genome-wide association study)	7	5	0	2	2	2	3	5	5	3	6	40
Scillitani 2004 [11] (Cross-sectional)	7	5	0	2	2	2	3	2	6	4	6	39
Shakhssalim 2010 [48] (Case-control)	6	4	4	2	2	2	2	3	3	4	4	36
Siomou 2017 [52] (Case-control)	4	4	3	2	2	5	2	2	2	2	4	32
Vezzoli 2014 [30] (Cross-sectional)	7	5	0	3	2	2	4	5	4	4	6	42
Vezzoli 2011 [31] (Case-control)	7	5	5	5	2	2	7	5	3	4	5	50
Vezzoli 2002 [32] (Case-control)	7	6	6	5	2	2	3	5	6	5	6	53
Wang 2017 [19] (Case-control)	7	5	5	2	2	5	7	6	7	4	6	56
Wang 2007 ^#^ [53] (Cross-sectional)	3	3	0	3	2	2	2	3	2	2	4	26
VDR												
Ames 1999 [13] (Cross-sectional)	7	5	0	4	2	2	2	4	5	3	6	40
Ferrari 1999 [49] (Cross-sectional)	4	5	0	3	2	4	3	4	5	2	4	36
Jehan 2010 [54] (Association study)	6	4	0	3	2	2	3	2	2	3	4	31
Nakano 2000 [12] (Case-control)	3	5	6	6	2	4	3	5	4	3	5	46
Relan 2004 [55] (Case-control)	4	4	4	3	2	2	3	3	1	2	5	33
Different Gene Polymorphisms												
Arcidiacono 2018 [33] (Cross-sectional)	7	5	0	4	2	5	4	6	6	6	7	52
Bellia 2016 [50] (Cross-sectional)	4	5	0	5	2	1	2	5	3	5	6	38
Fujita 2013 [56] (Cross-sectional)	4	2	0	2	1	1	2	2	2	3	4	23
Gianfagna 2013 [34] (Cross-sectional)	6	5	0	3	2	4	4	6	6	6	6	48
Howles 2019 [35] (Genome-wide association study)	7	4	0	6	2	3	4	5	5	6	7	49
Hwang 2014 [36] (Case-control)	7	4	4	5	4	4	2	6	7	4	7	54
Kim 2018 [37] (Cross-sectional)	7	4	0	7	4	6	7	6	7	6	7	61
Koek 2010 [38] (Cross-sectional)	7	5	0	6	4	3	5	6	6	6	7	55
Masi 2007 [51] (Cross-sectional)	7	6	0	4	2	1	1	6	2	3	7	39
Shakhssalim 2014 [47] (Case-control)	7	6	6	5	2	1	1	6	2	2	7	45

^#^ only available as abstracts. Studies with control groups: poor quality ≤35 (red), >moderate quality 35 and ≤45 (yellow), good quality >45 (green); studies without control groups: poor quality ≤32 (red), moderate quality >32 and ≤40 (yellow), good quality >40 (green). Abbreviations: CASR: calcium-sensing receptor; VDR: vitamin D receptor.

**Table 2:** Characteristics of studies assessing polymorphisms of the calcium sensing receptor (CASR).

Study Reference Country Study Design	Description of Study Population	SNPs Associated with Phenotype (Gene, rs#, Nucleotide Change, Amino Acid Change)	Frequency of SNP in Study Population	Description of SNP and Phenotype	Association of SNP with Calcium Change
Annerbo 2015 ^#^ [8] Sweden Cross-sectional	N = 1016 (515 males, 501 females) **Age**: 70 years **Ethnicity:** N/A **Inclusion:** healthy individuals **Exclusion:** N/A	*CASR* rs1801725 (NCBI G/T, A986S, Ala > Ser) *CASR* rs1042636 (NCBI A/G, R990G, Arg > Gly) *CASR* rs1801726 (NCBI G/A,C, Q1011E)	Carriers of T/G A/A G/C, present in 2% of the cohort, G/G A/A C/C present in 52.3% of the cohort	Small but significant changes in calcium level for carriers of the T allele in rs1801725	Carriers of the T allele in rs1801725 had significantly higher serum calcium levels 2.38 vs. 2.36 mmol/L (9.54 vs. 9.46 mg/dL), *p* = 0.016, carriers of T/G A/A G/C had significantly higher serum calcium compared to the most common haplotype G/G A/A C/C 2.37 vs. 2.36 mmol/L (9.50 vs. 9.46 mg/dL), *p* < 0.001
Arcidiacono 2009 ^#^ [10] Italy Case-control	N = 296 cases (155 kidney stone formers, 141 non-stone formers), 453 controls **Age**: N/A **Ethnicity:** N/A **Inclusion:** primary hyperparathyroidism (PHPT) patients as cases, healthy controls **Exclusion:** N/A	*CASR* rs1042636 (NCBI A/G, R990G, Arg > Gly) *CASR* rs7652589 (NCBI A/G) *CASR* rs4678013 (NCBI T/G) *CASR* rs1501899 (NCBI A/G,T)	Allele frequency was not significantly different in PHPT patients and controls. 990G variant allele more frequent in stone-forming than non-stone-forming patients (7.4% vs. 1.8%, RR = 4.4, *p* = 0.001)	SNPs/haplotype modifying *CASR* gene promoter activity favour stone formation in PHPT patients and reduce *CASR* expression in parathyroid and kidney tubular cells	Patients carrying GGG/GGG had higher serum ionized calcium than patients with any other haplotypes 1.50 ± 0.178 vs. 1.46 ± 0.122 mmol/L (6.01 ± 0.71 vs. 5.85 ± 0.489 mg/dL), *p* = 0.04. PHPT patients carrying one or two copies of 990 G variant allele had higher renal calcium excretion than homozygotes for 990R allele, *p* = 0.035
Bochud 2011 ^#^ [39] Switzerland Cross-sectional	N = 5319 **Age:** N/A (adults) **Ethnicity:** Caucasian **Inclusion:** participants from the population-based CoLaus study **Exclusion:** N/A	*CASR* rs11716910 (A/G)	N/A	Association of SNP with albumin-corrected serum calcium levels in the absence, but not in the presence, of calcium and/or vitamin D supplements	*CASR* SNP most strongly associated with serum calcium: 2.22 mmol/L (8.90 mg/dL) for AA, 2.21 mmol/L (8.86 mg/dL) for AG and 2.20 mmol/L (8.82 mg/dL) for GG genotype, *p* for trend < 0.0001
Cerani 2019 [9] Cohorts from UK, USA, Europe, China Mendelian randomisation study	N = 61,079 **Age:** 18.9–76.4 years (mean age in cohorts) **Ethnicity:** mixed (Northern European, North western European, European American, Southern Chinese) **Inclusion:** N/A **Exclusion:** N/A	*CASR* rs1801725 (NCBI G/T, A986S, Ala > Ser) *DGKD* rs1550532 (NCBI C/A,G) *GATA3* rs10491003 (NCBI C/G,T) *CARS* rs7481584 (NCBI G/A) *DGKH, VWA8* rs7336933 (NCBI G/A) *CYP24A1* rs1570669 (NCBI A/C,G,T) *VKORC1L1* rs17711722 (NCBI C/T)	Ca increasing allele and allele frequency: *CASR* (T) 0.15; *DGKD* (C) 0.32; *GATA3* (T) 0.09; *CARS* (G) 0.72; *DGKH*, *VWA8* (G) 0.85; *CYP24A1* (G) 0.34; *VKORC1L1* (T) 0.47	Genetic predisposition to increased serum calcium levels in individuals with normal calcium levels is not associated with an increase in estimated bone mineral density and does not provide clinically relevant protection against fracture	Serum calcium effect for calcium-increasing allele: *CASR* 0.0178 mmol/L (0.0713 mg/dL), *p* = 9 × 10^−86^; *DGKD* 0.0045 mmol/L (0.0180 mg/dL), *p* = 8 × 10^−11^; nearby *GATA3* 0.0068 mmol/L (0.0273 mg/dL), *p* = 5 × 10^−9^; *CARS* 0.0045 mmol/L (0.0180 mg/dL), *p* = 1 × 10^−10^; *DGKH*, *VWA8* 0.0055 mmol/L (0.0220 mg/dL), *p* = 9 × 10^−10^; near *CYP24A1* 0.0045 mmol/L (0.0180 mg/dL), *p* = 9 × 10^−12^; near *VKORC1L1* 0.00375 mmol/L (0.0150 mg/dL), *p* = 8 × 10^−9^
Chang 2015 [23] USA Genome-wide association study (GWAS)	N = 9034 (4765 males, 4269 females) **Age:** 9.9 years **Ethnicity:** European-American and African-American **Inclusion:** N/A **Exclusion:** N/A	*CASR* rs1801725 (T/G, A986S, Ala > Ser) *GCKR* rs780094 (A/G) *GATA3* rs10491003 (A/G)	N/A	*CASR* mediated calcium regulation in both African-American and European-American children and association of calcium level with *GCKR* and *GATA3* in European-American children	African-American children: association of *CASR* SNP with serum calcium level for minor allele T; European-American children: association of SNP with serum calcium level for *CASR* SNP for minor allele T, *GCKR* SNP with minor allele A, and *GATA3* with minor allele A
Cole 1999 [40] Canada Cross-sectional	N = 163 females **Age:** 18–35 years **Ethnicity:** Caucasian **Inclusion:** healthy women **Exclusion:** women who were not white and whose relatives were already enrolled in the parent study	*CASR* rs1801725 (NCBI G/T, A986S, Ala > Ser)	115 (70.6%) were homozygous for GG (AA) genotype, 43 (26.3%) women were heterozygous for GT (AS) genotype and 5 (3.1%) were homozygous for TT (SS) genotype	The *CASR* A986S variant has a significant effect on extracellular calcium	Significant correlation between *CASR* genotype and serum calcium corrected for albumin: AS heterozygotes 2.45 ± 0.02 mmol/L (9.82 ± 0.08 mg/dL) vs. AA homozygotes 2.38 ± 0.01 mmol/L (9.54 ± 0.04 mg/dL), *p* = 0.013, no difference between SS 2.45 ± 0.04 mmol/L (9.81 ± 0.16 mg/dL) and AS genotype; no differences between genotypes for total serum calcium
Cole 2001 [41] Canada Cross-sectional	N = 387 females **Age:** 18–35 years **Ethnicity:** Caucasian **Inclusion:** healthy women **Exclusion:** subjects reporting other than Caucasian ethnicity and relatives already in the parent study	*CASR* rs1801725 (NCBI G/T, A986S, Ala > Ser)	Frequencies of TT (SS), GT (AS), and GG (AA) genotypes were 6 (1.6%), 107 (27.6), and 274 (70.8%), respectively	Significant association between a common *CASR* A986S polymorphism and concentrations of a serum electrolyte	Mean serum calcium was significantly greater in AS heterozygotes 2.36 ± 0.01 mmol/L (9.45 ± 0.05 mg/dL), *p* = 0.002, or SS homozygotes 2.46 ± 0.07 mmol/L (9.88 ± 0.29 mg/dL), *p* = 0.015, in comparison to those with the AA homozygous genotype 2.30 ± 0.01 mmol/L (9.23 ± 0.04 mg/dL)
Corebetta 2006 [42] Italy Case-control	N = 94 cases (14 males, 80 females), 137 controls **Age:** 66 ± 12 years **Ethnicity:** Caucasian **Inclusion:** cases: PHPT patients, controls: age- and sex-matched, Caucasians without personal and family history of kidney stones, normal serum creatinine, calcium and phosphate, and 24 h urinary calcium levels, and not taking any drugs **Exclusion:** N/A	*CASR* rs1042636 (NCBI A/G R990G, Arg > Gly)	*CASR* R909G cases (*n* = 94): RR *n* = 83, RG *n* = 9, GG *n* = 2; controls (*n* = 137): RR *n* = 128, RG *n* = 8, GG *n* = 1	24 h urinary calcium was significantly higher in R/G + G/G vs. R/R patients with PHPT	Cases: lower 24 h urinary calcium (mmol/24 h) for RR genotype (6.77 ± 4.31) vs. RG + GG (9.05 ± 2.05), *p* = 0.012, no differences between genotypes for ionized calcium or serum calcium levels Controls: no data
Guha 2015 [24] India Case-control	N = 400 (255 males, 145 females) **Age:** 39.93 ± 11.73 years (range 18–60 years) **Ethnicity:** Indian **Inclusion:** cases: patients with at least one calcium kidney stone, controls: age- and sex- matched healthy subjects without personal and familial history of kidney stone, normal serum creatinine and calcium concentrations and no evidence of diseases at physical examination **Exclusion:** patients taking any drug affecting electrolyte or citrate handling (steroids, vitamin D, etc.), with endocrine or other disorders in addition to stone disease, abnormal serum creatinine, abnormal serum electrolyte concentration, recurrent urinary infections, evidence of cystic disorders of the kidney and nephropathy etc.	*CASR* rs1801725 (G/T, A986S, Ala > Ser) *CASR* rs1042636 (A/G, R990G, Arg > Gly)	*CASR* rs1801725: GG *n* = 278 (*n* = 116 cases, *n* = 162 controls), GT *n* = 119 (*n* = 82 cases, *n* = 37 controls), TT *n* = 3 (*n* = 2 cases, *n* = 1 controls); *CASR* rs1042636: AA *n* = 216 (*n* = 86 cases, *n* = 130 controls), AG *n* = 168 (*n* = 99 cases, *n* = 69 controls), GG *n* = 16 (*n* = 15 cases, *n* = 1 controls)	Common variants in *CASR* were associated with kidney stone disease in the eastern part of India	Higher serum calcium levels mmol/L (mg/dL) for *CASR* rs1801725 (Ala986Ser) GT + TT genotype 2.43 ± 0.05 cases, 2.41 ± 0.06 controls (9.72 ± 0.18, 9.64 ± 0.25 respectively) compared with GG genotype 2.30 ± 0.05 cases, 2.35 ± 0.05 controls (9.20 ± 0.20, 9.45 ± 0.21 respectively), *p* < 0.001, higher urinary calcium excretion (mmol/24 h) for rs1042636 (Arg990Gly) AG+GG genotype compared with AA genotype only for cases (8.10 ± 0.51 vs. 7.58 ± 0.57, *p* < 0.001)
Jorde 2013 [43] Norway Case-control	N = 9404 (4448 males, 4956 females) **Age:** 59.6 ± 13.7 years (in 1994) **Ethnicity:** N/A **Inclusion:** selected participants from the Tromsø Study 1994 and randomly selected controls **Exclusion:** N/A	*CASR* rs17251221 (A/G)	Genotype frequency *CASR* rs17251221: AA *n* = 7467 (79.4%), GA *n* = 1829 (19.5%), GG *n* = 108 (1.1%)	The minor GG homozygote genotype (high serum calcium) had a significant twofold increased risk (HR 2.32, 95% CI 1.24, 4.36) for prostate cancer compared to the major AA genotype	Significant association of *CASR* rs17251221 with serum calcium levels mmol/L (mg/dL) between AA genotype 2.38 ± 0.10 (9.54 ± 0.40) vs. GG genotype 2.42 ± 0.11(9.70 ± 0.44), *p* < 0.001, but not GA genotype 2.40 ± 0.10 (9.62 ± 0.40)
Jung 2009 [25] USA Population-and family-based association study	N = 106 (49 males, 57 females) **Age:** 18–36 years **Ethnicity:** African-American **Inclusion:** normotensive and in good health, none were taking medication with the exception that some of the female subjects used oral contraceptives **Exclusion:** N/A	*CASR* rs6438712 (A/G) *CASR* rs4678172 (G/T) *CASR* rs9874845 (A/T) *CASR* rs4678059 (A/T) *CASR* rs1965357 (C/T) *CASR* rs937626 (A/G)	Minor allele frequency: rs6438712 A 0.28; rs4678172 T 0.29; rs9874845 A 0.31; rs4678059 A 0.12; rs1965357 G 0.15; rs937626 G 0.34	Functional heterogeneity in *CASR* may affect the level of blood pressure in African-Americans. SNPs associated with urinary calcium excretion in both population- and family-based association studies	From 15 *CASR* SNPs examined, 6 were associated with lower urinary calcium (uCa) excretion. Calcium excretion rate, log uCa (mg/h) for participants carrying the minor allele: rs6438712 A 0.03; rs4678172 T 0.02; rs9874845 A 0.02; rs4678059 A < 0.0001; rs1965357 C 0.0007; rs937626 G 0.02 (*p* ≤ 0.001)
Kapur 2010 [26] Switzerland Meta-analysis of genome-wide association study	N = 12,865 **Age:** 50.7–70.4 years **Ethnicity:** European and Indian Asian descent **Inclusion:** participants from the CoLaus study (population-based sample from Lausanne, Switzerland), the London Life Sciences Prospective Population Study (LOLIPOP, population-based cohort study), the InCHIANTI study (population-based epidemiological study), the Baltimore longitudinal study on Aging (BLSA, population-based study), the deCODE study **Exclusion:** N/A	*CASR* rs1801725 (NCBI G/T, A986S, Ala > Ser)	T allele frequencies 16.76% in European and 19.98% Indian Asian cohorts	Common *CASR* variants modulate serum calcium levels in the adult general population. The rs1801725 T allele (A986S) is associated with higher serum calcium, but no significant association between rs1801725 and the calcium-related outcomes (coronary heart disease, myocardial infarction, hypertension, stroke, osteoarthritis, osteoporosis and kidney stones)	*CASR* rs1801725 explains 1.26% of the variance in serum calcium. At an average serum calcium level of 2.25 mmol/L (9.02 mg/dL), each rs1801725 T allele yields an increase of 0.01874 mmol/L (0.0751 mg/dL), or 21% of one standard deviation of serum calcium levels in a normal population.
Laaksonen 2009 [27] Finland Cross-sectional	N = 350 (135 males, 215 females) **Age:** 30–42 years **Ethnicity:** Caucasian **Inclusion:** healthy adults, subgroup from the population-based FINRISK survey **Exclusion:** N/A	*CASR* rs1801725 (NCBI G/T, A986S, Ala > Ser)	Male (*n* = 104): AA *n* = 82 (79%), AS *n* = 19 (18%), SS *n* = 3 (3%); female (*n* = 182): AA *n* = 143 (78.5%), AS *n* = 36 (20%), SS *n* = 3 (1.5%)	The *CASR* 986S allele was associated with higher serum ionized calcium. Vitamin D receptor (VDR), *CASR* and parathyroid hormone (PTH) polymorphisms contribute to the genetic regulation of calcium homeostasis and peripheral bone density	Higher serum ionized calcium mmol/L (mg/dL) for individuals with AS or SS genotype (mean: 95% CI, both sexes): AA 1.200: 1.196–1.204 (4.809: 4.793–4.825, AS 1.214: 1.206–1.222 (4.866: 4.833–4.898), SS 1.239: 1.214–1.264 (4.966: 4.866–5.066) (adjusted for BMI, S-25OHD and calcium intake, ANOVA, *p* < 0.001)
Larsson 2017 [28] Sweden Mendelian randomization study	Up to 60,801 coronary artery disease (CAD) cases (approximately 70% with myocardial infarction) and 123,504 non-cases from 48 cohort and case-control studies **Age:** 45–75 years **Ethnicity:** European (77%), South Asian (13%), and East Asian (6%) ancestry **Inclusion:** case status was determined using a broad definition of CAD, including myocardial infarction (approximately 70% of the total number of cases), acute coronary syndrome, chronic stable angina, or coronary artery stenosis greater than 50% **Exclusion:** N/A	*CASR* rs1801725 (NCBI G/T, A986S, Ala > Ser) *CYP24A1* rs1570669 (NCBI A/C,G,T) *DGKD* rs1550532 (NCBI C/A,G) *CARS* rs7481584 (NCBI G/A) *DGKH/KIAA0564* rs7336933/NCBI G/A) *GATA3* rs10491003 (NCBI C/G,T)	Frequency of the calcium-raising allele: *CASR* (rs1801725): T 0.14 *CYP24A1* (rs1570669): G 0.36 *DGKD* (rs1550532): C 0.31 *CARS* (rs7481584): G 0.69 *DGKH/KIAA0564* (rs7336933): G 0.86 *GATA3* (rs10491003): T 0.09	A genetic predisposition to higher serum calcium levels was associated with increased risk of CAD and myocardial infarction. Meta-analysis (combining the 6 SNPs): 0.5 mg/mL increase in serum calcium levels resulted in OR 1.25 (95% CI 1.08–1.45; *p* = 0.003) for CAD and OR 1.24 (95% CI 1.05–1.46; *p* = 0.009) for myocardial infarction	Serum calcium level increased by mmol/L (mg/dL) per additional: T allele for *CASR* (rs1801725): 0.018 (0.071) G allele for *CYP24A1* (rs1570669): 0.005 (0.018) C allele for *DGKD* (rs1550532): 0.005 (0.018) G allele for *CARS* (rs7481584): 0.005 (0.018) G allele for *DGKH/KIAA0564* (rs7336933): 0.006 (0.022) T allele for *GATA3* (rs10491003): 0.006 (0.022), *p* < 0.001
Lorentzon 2001 [44] Sweden Association study	N = 97 females **Age:** 16.9 ± 1.2 years **Ethnicity:** Caucasian **Inclusion:** at least 2 years post menarche, no disease or medication known to affect bone metabolism **Exclusion:** N/A	*CASR* rs1801725 (NCBI G/T, A986S, Ala > Ser)	Subjects without S *n* = 69, with S *n* = 28, genotype frequencies: AA 71%, AS 26%, SS 3%	Subjects with the S allele had higher levels of plasma calcium (corrected for albumin) and lower bone mineral density at the lumbar spine compared with subjects lacking the S allele	Plasma calcium corrected for albumin mmol/L (mg/dL): 2.14 ± 0.06 (8.58 ± 0.24) for subjects without S (*n* = 68) vs. 2.17 ± 0.06 (8.70 ± 0.24) for subjects with S (*n* = 26), *p* < 0.05, no difference in plasma calcium between genotypes
Majid 2015 [45] Pakistan Cross-sectional	N = 140 females **Age:** 18–38 years **Ethnicity:** N/A **Inclusion:** adult premenopausal females with known vitamin D deficiency (VDD), intact parathyroid hormone (iPTH), and calcium levels **Exclusion:** medical disorder of bone minerals, such as PHPT or hypoparathyroidism, any medications known to affect calcium metabolism, such as calcium tablets or bisphosphonate, and vitamin D supplements, individuals on hormonal therapy, and pregnant females	*CASR* rs1801725 (NCBI G/T, A986S, Ala > Ser) *CASR* rs1042636 (A/G, R990G, Arg > Gly)	Frequency of A986S alleles: GG 68%, GT 25%, TT 7% R990G alleles: AA 80%, AG 8.9%, GG 11.1%	Patients with VDD with the GG genotype of R990G SNP are prone to have higher iPTH levels and lower calcium compared with AG and AA genotype subjects	Serum calcium level mmol/L (mg/dL) significantly associated with *CASR* rs1042636 (R990G) genotype: AA 2.3 ± 0.1 (9.1 ± 0.3) (*n* = 112), AG 2.3 ± 0.1 (9.1 ± 0.3) (*n* = 12), GG 2.1 ± 0.1 (8.5 ± 0.4) (*n* = 16), *p* = 0.002, no association for *CASR* A986S genotypes
März 2007 [29] Germany Case-control	N = 3259 (2280 males, 979 females) **Age:** cases (CAD): males 63 ± 10, females 66 ± 10, controls (no CAD): males 55 ±1 2, female 62 ± 10 years **Ethnicity:** Caucasian **Inclusion:** white patients hospitalized for coronary angiography from the Ludwigshafen Risk and Cardiovascular Health (LURIC) study **Exclusion:** acute illness other than acute coronary syndromes, chronic noncardiac diseases, and a history of malignancy within the five past years	*CASR* rs1801725 (NCBI G/T, A986S, Ala > Ser)	Controls (no CAD): AA *n* = 517 (74%), AS *n* = 168 (24%), SS *n* = 13 (2%) Cases (CAD): AA *n* = 1799 (70%), AS *n* = 6699 (27%), SS *n* = 63 (3%); CAD + myocardial infarction (MI): AA *n* = 9407 (69%), AS *n* = 378 (28%), SS *n* = 40 (3%)	*CASR* polymorphism affects calcium homeostasis and is associated with CAD, MI, all cause, and cardiovascular mortality	Carrier of S allele (AS or SS) had significantly higher calcium levels, *p* < 0.001, and albumin-adjusted calcium levels, *p* < 0.001, compared with AA homozygotes, independent of presence or absence of CAD
O’Seaghdha 2010 [46] N/A Genome-wide association studies	N = 20,611 (46.8% males, 69.4% females) **Age:** 43.6 ± 9.8 to 77 ± 5.4 years **Ethnicity:** individuals of European ancestry **Inclusion:** participants from 6 studies: the family-based Framingham Heart Study (FHS), the prospective population-based Atherosclerosis Risk in Communities (ARIC) study, the Cardiovascular Health Study (CHS), population-based Rotterdam Study (RS), the community-based prospective cohort study Health ABC (HABC), the Age Gene/Environment Susceptibility (AGES)-Reykjavik Study **Exclusion:** N/A	*CASR* rs17251221 (A/G)	Minor allele G frequency: 14%	Variation in *CASR* influences serum calcium concentration. G allele of rs17251221 also associated with higher serum magnesium levels, lower serum phosphate levels and lower bone mineral density at the lumbar spine	SNP is associated with 0.015 mmol/L (0.06 mg/dL) higher serum calcium levels per copy of the minor G allele and accounted for 0.54% of the variance in serum calcium concentrations
Scillitani 2004 [11] Italy Cross-sectional	N = 377 (184 males, 137 premenopausal, 56 postmenopausal females) **Age:** men median age (range): 43 years (18–65), premenopausal women: 37 years (18–52), postmenopausal women: 53 years (48–65) **Ethnicity:** Caucasian **Inclusion:** Caucasian healthy adults retrospectively recruited from a blood donor clinic **Exclusion:** taking medication or known to be affected by diseases affecting calcium metabolism	*CASR* rs1801725 (NCBI G/T, A986S, Ala > Ser) *CASR* rs1042636 (NCBI A/G, R990G, Arg > Gly) *CASR* NCBI rs1801726 (G/A,C, Q1011E, Gln > Glu)	Genotype frequency: A986S: AA *n* = 223, AS *n*= 126, SS *n* = 27 R990G: RR *n* = 345, RG *n* = 31 Q1011E: QQ *n* = 335, QE *n* = 21	Association of increased ionized calcium with allele variants of *CASR*	A986S: common AA genotype had significantly lower ionized calcium mmol/L (mg/dL) than subjects with one or two S alleles 1.221 ± 0.003 vs. 1.239 ± 0.003 (4.894 ± 0.012 vs. 4.966 ± 0.012), *p* = 0.0001 R990G: common RR (AA) genotype had higher ionized calcium mmol/L (mg/dL) than those with 990G allele 1.230 ± 0.002 vs. 1.213 ± 0.007 (4.930 ± 0.008 vs. 4.862 ± 0.028), *p* = 0.032 Q1011E: common QQ (CC) genotype had lower ionized calcium mmol/L (mg/dL) than those with QE (CG) genotype 1.227 ± 0.002 vs. 1.255 ± 0.008 (4.918 ± 0.008 vs. 5.030 ± 0.032), *p* = 0.002
Shakhssalim 2010 [48] Iran Case-control	N = 206 males (99 cases, 107 controls) **Age:** 30–55 years **Ethnicity:** N/A **Inclusion:** cases: idiopathic recurrent calcium kidney stone-forming men without any systematic disorders, controls: healthy volunteer men **Exclusion:** cases: history of known metabolic, gastrointestinal, hepatic, renal or endocrinological diseases	*CASR* rs1801725 (NCBI G/T, A986S, Ala > Ser) *CASR* rs1042636 (NCBI A/G, R990G, Arg > Gly) *CASR* rs1801726 (NCBI G/A,C, Q1011E, Gln > Glu)	A989S cases: AA *n* = 71 (71.7%), AS *n* = 26 (26.3%), SS *n* = 2 (2%), controls: AA *n* = 93 (86.9%), AS *n* = 14 (13.1%), SS *n* = 0 (0%) R990G cases: RR *n* = 87 (87.9%), RG *n* = 10 (10.1%), GG *n* = 2 (2%), controls: RR *n* = 105 (98.1%), RG *n* = 2 (1.9%), GG *n* = 0 (0%) E1011Q cases: EE *n* = 0 (0%), EQ *n* = 5 (5.1%), QQ *n* = 94 (94.9%), controls: EE *n* = 0 (0%), EQ *n* = 0 (0%), QQ *n* = 107 (100%)	986S, 990G and 1011Q alleles were associated with a recurrent calcium kidney stone-forming state. 986S and 1011Q alleles, but not 986S, were associated with hypercalcemia	Cases and controls combined. R990G: RR genotype showed higher serum ionized calcium mmol/L (mg/dL) compared with RG or GG 1.15 ± 0.04 vs. 1.13 ± 0.03 (4.62 ± 0.18 vs. 4.52 ± 0.10), *p* = 0.01; Q1011E: QQ showed lower total serum calcium mmol/L (mg/dL) compared with EQ 2.41 ± 0.15 vs. 2.60 ± 0.15 (9.67 ± 0.60 vs. 10.44 ± 0.59), *p* = 0.01; R990G and Q1011E: no difference in mean calcium 24 h urine (mg/24 h) between genotypes; A989S: no difference between wild type and mutant for serum calcium or 24 h urinary calcium excretion
Siomou 2017 [52] Greece Case-control	N = 112 (50 cases, 62 controls) **Age:** children **Ethnicity:** N/A **Inclusion:** cases: children with idiopathic hypercalciuria, controls: age-and gender matched **Exclusion:** N/A	*CASR* rs1801725 (G/T, A986S, Ala > Ser) *CASR* rs1042636 (A/G, R990G, Arg > Gly) *CASR* rs1801726 (C/G, Q1011E, Gln > Glu)	A986S: minor T allele higher in cases than controls (30% vs. 10%, *p* = 0.0001), GT/ΤΤ higher in cases than controls (54% vs. 19%, *p* = 0.00015); R990G: minor G allele higher in cases than controls (6% vs. 2%, *p* = 0.058), GA genotype higher in cases than controls (12% vs. 3%, *p* = 0.054); Q1011E: SNPs frequency similar in both cases and controls	Polymorphisms A986S and R990G of the *CASR* gene were associated with idiopathic hypercalciuria but not with the severity of hypercalciuria	Positive association in the T allele with renal calcium excretion, independent of age and serum levels of calcium, intact parathormone and 25-hydroxy-vitamin D (beta: 0.12, 95% CI 0.05–0.33, *p* < 0.0001), no results reported for rs1042636 and rs1801726
Vezzoli 2014 [30] Italy Observational (cross-sectional)	N = 296 (48 males, 248 females; 155 PHPT patients with stones, 141 without stones) **Age:** PHPT patients with stone 56 ± 1.1 years, without stone 63 ± 1.1 years **Ethnicity:** N/A **Inclusion:** patients with sporadic PHPT **Exclusion:** patients with biochemical findings or a family history suggesting FHH, or with parathyroid carcinoma, or taking drugs or having diseases influencing bone metabolism other than PHPT	*CASR* rs1501899 (G/A) *CASR* rs1042636 (NCBI A/G, R990G, Arg > Gly)	Minor allele frequency for rs1501899 (AA or GA): PHPT patients with stones *n* = 95 (61.3%), without stones *n* = 67 (47.5%); Arg990Gly (Gly): PHPT patients with stones *n* = 22 (14.2%), without stones *n* = 5 (3.5%)	Serum ionized calcium and calcium excretion were higher in patients carrying the minor allele in both SNPs compared with wild-type. Rs1501899 and Arg990Gly SNPs may predispose PHPT patients to nephrolithiasis and to a more severe phenotype of PHPT	Patients carrying the minor alleles in both SNPs rs1501899 (AA or GA) and rs1042636 (990Gly/990Arg or 990Gly/990Gly had higher serum ionized calcium 1.59 ± 0.072 mmol/L (6.37 ± 0.289 mg/dL) compared with patients carrying wild-type alleles at both SNPs 1.47 ± 0.012 mmol/L (5.89 ± 0.048 mg/dL), *p* = 0.03, and higher urine calcium (µmol/mmol creatinine) 231 ± 37.5 vs. 151 ± 9.6, *p* = 0.05
Vezzoli 2011 [31] Ital Case-control, genotype–phenotype association study	N = 332 PHPT patients (62 males, 270 females), 453 healthy controls (180 males, 273 females) **Age:** 30–55 years **Ethnicity:** N/A **Inclusion:** patients with sporadic PHPT, controls: healthy volunteers who had a normal clinical examination **Exclusion:** patients with hormonal and biochemical findings or a family history suggesting FHH, patients with parathyroid carcinoma, low serum creatinine of 1.2 mg/dL or less	*CASR* rs7652589 (G/A) *CASR* rs1501899 (G/A)	Allele frequency of rs7652589 A controls: 33.4%, PHPT patients: 32.2%, rs7652589 G controls: 66.6%, PHPT patients: 67.8% rs1501899 A controls: 31.5%, PHPT patients: 32.1%, rs1501899 G controls: 68.5%, PHPT patients: 67.9% haplotype including both SNPs AA controls: 30.4%, PHPT patients: 31.5%, AG and GA controls: 4.3%, PHPT patients: 2%, GG controls: 65.3%, PHPT patients: 66.6%	PHPT patients with AA/AA or AA/GG diplotype had higher serum concentrations of ionized calcium than patients with GG/GG diplotype. SNPs in the regulatory region of *CASR* gene may increase the risk for kidney stone formation in PHPT patients	Higher serum ionized calcium mmol/L (mg/dL) for PHPT patients with diplotype AA/AA or AA/GG vs. GG/GG 1.50 ± 0.015 vs. 1.47 ± 0.011 (6.01 ± 0.060 vs. 5.89 ± 0.044), *p* = 0.04, and higher urine calcium (µmmol/mmol creatinine) for diplotype AA/AA or AA/GG vs. GG/GG (183 ± 12.2 vs. 150 ± 11.4, *p* = 0.049)
Vezzoli 2002 [32] Italy Case-control	N = 97 normocalciuric stone formers (65 males, 32 females), 134 hypercalciuric stone formers (78 males, 56 females), 101 normocalciuric healthy controls (58 males, 43 females) **Age:** normocalciuric stone formers 45 ± 1.5 years, hypercalciuric stone formers 47 ± 1.1 years, healthy controls 46 ± 1.2 years **Ethnicity:** White subjects **Inclusion:** stone formers: at least one calcium kidney stone, normal plasma creatinine, sodium, potassium, and calcium concentrations, stones in urinary tract smaller than 5 mm in diameter; hypercalciuric: 24-h calcium excretion greater than 7.5 mmol in male patients or 6.25 mmol in female patients or greater than 0.1 mmol/kg of body weight independently of gender; controls: selected from the InCHIANTI study, absence of hypercalciuria, age between 20 and 60 years, no clinical diagnosis of hypertension, diabetes, dyslipidemia, stroke, coronary heart disease, kidney stones, and no treatment with drugs affecting calcium metabolism, like calcium salts or vitamin D **Exclusion:** Stone formers: obstructive nephropathy or urinary tract dilatation, any treatment for kidney stones (thiazide, citrate, or others) for at least 3 months before the study, other diseases than kidney stone, any long-term medications	*CASR* rs1801725 (NCBI G/T, A986S, Ala > Ser) *CASR* rs1042636 (NCBI A/G, R990G, Arg > Gly) *CASR* rs1801726 (NCBI G/C, Q1011E, Gln > Glu)	Stone formers and healthy controls Group 1 *n* = 133 (57.6%): Ala986, Arg990, and Gln1011 homozygotes Group 2 *n* = 74 (32.0%): 986Ser homozygotes and Ala986Ser heterozygotes Group 3 *n* = 15 (6.5%): 990Gly homozygotes and Arg990Gly heterozygotes Group 4 *n* = 9 (3.9%): Gln1011Glu heterozygotes	Calcium excretion was higher in subjects bearing haplotype 3 and haplotype 3 explained 4.1% of the total variance of renal calcium excretion (multiple regression)	Higher urinary calcium excretion (mmol/24 h) in group 3 (9.18 ± 0.95) in stone formers and healthy controls compared with group 1 (6.05 ± 0.22), group 2 (6.44 ± 0.35), and group 4 (5.05 ± 0.86) (*p* = 0.003); no differences in plasma calcium between groups; higher urinary calcium excretion (mmol/24 h) in group 3 (9.68 ± 0.87) vs. group 1 (6.91 ± 0.26) in stone formers (*p* = 0.005), but no other groups, no differences in plasma calcium between groups
Wang 2017 [19] USA Retrospective case-control study, genome-wide association study	N = 583 females (199 cases, 384 age-matched controls) **Age:** 54.9 ± 4.4 years **Ethnicity:** African American and Caucasian **Inclusion:** breast cancer cases: defined from databases using ICD-9 code 174 (neoplasms of the female breast), tumour registries, calcium assay data, gender (= female), race (= Caucasian or African American) and genome-wide association studies (GWAS) genotyping data; controls: age-matched records with calcium and GWAS data, no evidence of any form of malignancy **Exclusion:** N/A	*CASR* rs1801725, (G/T, A986S, Ala > Ser)	G/G genotype (A986A) *n* = 458 (79%); G/T genotype (A986S) (20%); T/T genotype (S986S) *n* = 10 (2%); similar distribution between controls and breast cancer cases	Mean circulating calcium levels significantly higher in all subjects expressing the G/T and T/T genotypes of *CASR* compared to the wild type receptor (G/G)	Higher circulating calcium levels mmol/L (mg/dL) in control and breast cancer subjects carrying G/T (AS) genotype (*n* = 115) 2.31 ± 0.12 (9.25 ± 0.48), *p* = 0.006, and T/T (SS) genotype (*n* = 10) 2.37 ± 0.13 (9.48 ± 0.50), *p* = 0.024, compared with G/G (AA) genotype (*n* = 458) 2.27 ± 0.13 (9.13 ± 0.51)
Wang 2007 ^#^ [53] China Cross-sectional	N = 202 females **Age:** 27 ± 5 years **Ethnicity:** Han Chinese **Inclusion:** healthy young women of Han nationality in Beijing area **Exclusion:** N/A	*CASR* rs1042636 (NCBI A/G, R990G, Arg > Gly)	Frequencies of genotypes for rs1042636 R990G: RR 21.3%, GR 51.0% and GG 27.7% (G allele more common)	R990G polymorphism was associated with serum calcium and subjects with R allele had higher levels of serum calcium	Significant differences in serum calcium mmol/L (mg/dL) for rs1042636 R990G: GG 2.44 ± 0.10 (9.78 ± 0.40), GR 2.46 ± 0.08 (9.86 ± 0.32) and RR 2.48 ± 0.08 (9.94 ± 0.32), *p* = 0.042; calcium adjusted by albumin mmol/L (mg/dL): GG 2.30 ± 0.10 (9.22 ± 0.40), GR 2.32 ± 0.09 (9.30 ± 0.36) and RR 2.32 ± 0.10 (9.30 ± 0.40), *p* = 0.02

^#^ only available as abstracts. Abbreviations: CAD: coronary artery disease; CARS: cysteinyl-tRNA synthetase CASR: calcium sensing receptor; CI: confidence interval; CYP24A1: cytochrome P450 family 24 subfamily A member 1; DGKD: diacylglycerol kinase delta; DGKH: diacylglycerol kinase eta; FHH: familial hypocalciuric hypercalcemia; GATA3: GATA binding protein 3; GCKR: glucokinase regulatory protein; GWAS: Genome-wide association study; iPHT: intact parathyroid hormone; MI: myocardial infarction; N/A: not available; OR: odds ratio; PHPT: primary hyperparathyroidism; PTH: parathyroid hormone; rs#: reference SNP ID; SNP: single nucleotide polymorphism; S-25OHD: serum 25-hydroxyvitamin D; VDD: vitamin D deficiency; VDR: vitamin D receptor; vs.: versus; VKORC1L1: vitamin K epoxide reductase complex subunit 1-like protein 1; VWA8 (KIAA0564): von Willebrand factor A domain-containing protein 8.

**Table 3:** Characteristics of studies assessing polymorphisms of the vitamin D receptor (VDR).

Study Reference Country Study Design	Description of Study Population	SNPs Associated with Phenotype (Gene, rs#, Nucleotide Change, Amino Acid Change)	Frequency of SNP in Study Population	Description of SNP and Phenotype	Association of SNP with Calcium Change
Ames 1999 [13] USA Cross-sectional	N = 72 (8 males, 64 females) **Age:** 7.5–12 years **Ethnicity:** 38 Caucasian, 18 African-American, 16 Mexican-American **Inclusion:** prepubertal and pubertal healthy children (Tanner stage 1–3), 5th and 95th percentiles for weight for age and height for age, diets containing ~1200 mg of calcium per day **Exclusion:** N/A Note: diet adapted to ~1200 mg of calcium per day 2 weeks prior to study and throughout the study period	*VDR Fok*1 rs2228570 (C/T)	Overall: 41.6% FF (wild type), 44.5% Ff, and 13.9% ff (mutant); Caucasians and Mexican-Americans: 33.3% FF, 48.2% Ff, 18.5% ff; African-American: 66.6% FF, 33.4% Ff	The FF wild type genotype was associated with greater calcium absorption (average 150 mg more calcium per day compared with the mutant ff genotype) and higher bone mineral density (BMD)	FF genotype (wild type) had 41.5% greater calcium absorption than ff (mutant) homozygotes, *p* = 0.04, and 17% greater calcium absorption than the Ff heterozygotes, *p* = 0.19
Ferrari 1999 [49] Switzerland Cross-sectional	N = 104 males **Age:** 24.3 ± 3.1 years (range 20.7–38.7 years) **Ethnicity:** Caucasian **Inclusion:** healthy subjects **Exclusion:** known acute or chronic diseases or medications that could affect intestinal absorption, kidney function, or bone turnover Note: 25 subjects (bb *n* = 15, BB *n* = 10) received first a low (calcium and phosphorus restriction through dietary counselling and intake of magnesium- and aluminium-containing phosphorus binder for 5 days), then a washout period followed by a high (consumption of products rich in calcium and phosphate plus additional 1000 mg phosphorus/day for 5 days) calcium-phosphorus diet for a total duration of 15 days.	*VDR Bsm*1 NCBI rs1544410 (A/G)	*Bsm*1: bb *n* = 46 (44%), Bb *n* = 37 (36%), BB *n* = 21 (20%)	Dietary modification of calcium-phosphate intake and *VDR Bsm*1 polymorphism were associated with alternations in fasting urinary calcium and renal calcium excretion	Influence of dietary modification for calcium (μmol/L glomerular filtration rate (GFR)) in fasting urine: baseline bb 0.025 ± 0.003 versus BB 0.020 ± 0.003, restriction bb 0.017 ± 0.001 vs. BB 0.017 ± 0.002, supplementation bb 0.022 ± 0.003 vs. BB 0.018 ± 0.002 (*p* = 0.04); and daily renal calcium excretion (mg/day): baseline bb 208 ± 14 vs. BB 198 ± 20, restriction bb 152 ± 8 vs. BB 128 ± 19, supplementation bb 198 ± 17 vs. BB 140 ± 15, *p* = 0.05, no influence on serum ionized calcium. No differences between *VDR Bsm*1 genotypes and serum ionized calcium, urinary calcium or daily calcium excretion without dietary modifications.
Jehan 2010 [54] Moldavia Association study	N = 204 (118 males, 86 females) **Age:** 7–16 years (boys 11.2 ± 1.7 years, girls 11.5 ± 1.8 years) **Ethnicity:** Caucasian **Inclusion:** healthy children and adolescents, calcium intake 493 mg/day including 69 mg/day as milk and dairy products **Exclusion:** children with known chronic diseases and/or body measures below or above 2 SDs	*VDR* rs4516035 (−1012 G/A)	GG: *n* = 43 (21.1%), GA: *n* = 90 (44.1%), AA: *n* = 71 (34.8%)	Height during growth is in part controlled by VDR expression and may be hampered in children and adolescents bearing a G/G genotype	Significant association of *VDR* rs4516035 with serum calcium mmol/L (mg/dL) levels (adjusted for serum protein levels): GG genotype 2.13 ± 0.05 (8.54 ± 0.20), GA genotype 2.24 ± 0.04 (8.98 ± 0.16), AA genotype 2.27 ± 0.04 (9.10 ± 0.16) (GG vs. AA, *p* = 0.0336, GG vs. GA, *p* = 0.0544)
Nakano 2000 [12] Japan Case-control	N = 247 (cases: 77 males, 70 females, controls: 54 males, 46 females) **Age:** cases 55 ± 10 years, controls 51 ± 17 years **Ethnicity:** Japanese **Inclusion:** cases: essential hypertension (systolic blood pressure > 160 mmHg and/or diastolic blood pressure > 95 mmHg); controls: normotensive (systolic blood pressure < 140 mmHg and/or diastolic blood pressure < 90 mmHg) no history of hypertension, not taken any antihypertensive medications or other medications that could affect blood pressure **Exclusion:** secondary causes of hypertension, diabetes mellitus, heart, liver, and kidney diseases	*VDR Bsm*1 NCBI rs1544410 (A/G)	Genotype frequency normotensives (*n* = 100): bb (mutant) 56%, Bb 36%, BB (wild type) 8%; hypertensives (*n* = 138): bb 60.1%, Bb 32.6%, BB 7.4%	BB (wild type) genotype of the *VDR* gene is associated with lower serum calcium levels, but not useful as a predictive marker for hypertension.	Lower serum total calcium mmol/L (mg/dL) for BB (wild type) genotype compared with mutant bb, *p* < 0.05. Normotensive: bb 4.5 ± 0.3 vs. BB 4.4 ± 0.5 (18.0 ± 1.2 vs. 17.6 ± 2.0), Bb 4.5 ± 0.4 (18.0 ± 1.6); hypertensives: bb 4.6 ± 0.3 vs. BB 4.4 ± 0.5 (18.4 ± 1.2 vs. 17.6 ± 2.0), Bb 4.6 ± 0.4 (18.4 ± 1.6). Lower serum ionized calcium mmol/L (mg/dL) for BB genotype compared with bb, *p* < 0.05. Normotensive: bb 1.17 ± 0.04 vs. BB 1.15 ± 0.04 (4.69 ± 0.16 vs. 4.61 ± 0.16), Bb 1.16 ± 0.04 (4.65 ± 0.16); hypertensives: bb 1.16 ± 0.04 vs. BB 1.14 ± 0.05 (4.65 ± 0.16 vs. 4.57 ± 0.20) (Bb 1.16 ± 0.04 (4.65 ± 0.16)
Relan 2004 [55] India Case-control	N = 250 (150 cases: 105 males, 45 females, 100 controls: 76 males, 24 females) **Age:** cases 39.38 ± 1.12 years (range 18–65 years), controls 43.25 ± 2.05 years **Ethnicity:** Indian **Inclusion:** cases: nephrolithiatic patients; controls: no evidence of stone disease and without any family history of stone disease **Exclusion:** N/A	*VDR Bsm*1 NCBI rs1544410 (A/G) *VDR Fok*1 rs2228570 (NCBI C/T)	Allele frequency of the *VDR Bsm*I restriction site: controls (*n* = 100): B wild type allele 60%, b mutant allele 40%; nephrolithiatic subjects (*n* = 150): B allele 52.7%, b allele 47.3%; hypercalciuric nephrolithiatic subjects (*n* = 47): B allele 38.3%, b allele 61.7%. *VDR Fok*1 restriction site: controls (*n* = 100): F wild type allele 55.4%, f mutant allele 44.4%; nephrolithiatic subjects (*n* = 150): F allele 40.9%, f allele 59.1%; hypercalciuric nephrolithiatic subjects (*n* = 47): F allele 56.63%, f allele 40.47%.	*VDR* polymorphisms may be associated with increased renal calcium excretion in hypercalciuric nephrolithiatic subjects (bb genotype and Ff and FF genotypes exhibit higher renal calcium excretion)	*VDR Bsm*1: significantly higher 24 h urinary calcium (mg/24 h) excretion, *p* = 0.001, in nephrolithiatic subjects with the bb homozygous mutant genotype (262.61 ± 24.28) compared with the Bb (165.76 ± 17.26) and homozygous BB (205.68 ± 14.29) genotypes. Hypercalciuric nephrolithiatic subjects, 24 h urinary calcium excretion significantly higher in bb genotype, *p* < 0.05, compared with Bb and BB genotypes. Serum calcium levels comparable in all genotypes in both nephrolithiatic and hypercalciuric nephrolithiatic subjects. *VDR Fok*1: nephrolithiatic subjects with homozygous (FF) or heterozygous (Ff) genotypes showed significantly higher, *p* < 0.05, calcium excretion compared to the ff genotype. Hypercalciuric nephrolithiatic subjects, heterozygotes (Ff) excrete more calcium than the ff genotype. Serum calcium was significantly higher, *p* < 0.05, in hypercalciuric nephrolithiatic subjects with the ff genotype compared with FF or Ff genotypes.

Abbreviations: BMD: bone mineral density; GFR: glomerular filtration rate; N/A: not available; rs#: reference SNP ID; SD: standard deviation; SNP: single nucleotide polymorphism; VDR: vitamin D receptor; vs.: versus.

**Table 4:** Characteristics of studies assessing different gene polymorphisms.

Study Reference Country Study Design	Description of Study Population	SNPs Associated with Phenotype (Gene, rs#, Nucleotide Change, Amino Acid Change)	Frequency of SNP in Study Population	Description of SNP and Phenotype	Association of SNP with Calcium Change
Arcidiacono 2018 [33] Italy Retrospective observational (cross-sectional)	N = 393 (317 males, 76 females) **Age:** 45 ± 10 years **Ethnicity:** N/A **Inclusion:**: never-treated, recently discovered patients with essential hypertension with high/normal blood pressure level or grade 1 or 2 hypertension **Exclusion:** patients with secondary causes of hypertension, endocrine disorders, body mass index > 32 kg/m^2^, or chronic and acute concomitant diseases (cardiocerebrovascular diseases, diabetes mellitus, or hepatic and kidney diseases) and women taking contraceptive pills	31 SNPs in the 3′ region of claudin-14 gene *CLDN14* rs219755 (G/A)	N/A	Claudin-14 genotype at the 39 region is associated with calcium excretion in 24-h urine	rs219755 (G/A) showed the strongest association with 24-h urinary calcium excretion (mg/24 h): AA 124 ± 73, GA 194 ± 100, GG 225 ± 124, *p* < 0.001
Bellia 2016 [50] Italy Cross-sectional	N = 393 (317 males, 76 females) **Age:** 60 ± 10.6 years **Ethnicity:** Caucasian **Inclusion:**: patients with one or more cardiovascular risk factor and asymptomatic for coronary vasculopathy **Exclusion:** any severe chronic disease, hepatic disease, chronic kidney disease (CKD), respiratory insufficiency, malignancy, and infectious disease	*AHSG* rs4918 (G/C, T256S, Thr > Ser)	GG (TT) 64%, GC (TS) 28%, CC (SS) 8%	Serum levels of Fetuin-A are linked to serum calcium homeostasis and *AHSG* genotype but not with coronary artery calcification (CAC) severity in subjects without coronary vasculopathy	Subjects carrying the CC (SS) genotype had lower levels of serum calcium mmol/L (mg/dL) 2.3 ± 0.03 (9.1 ± 0.1) compared with GG (TT) 2.3 ± 0.1 (9.4 ± 0.5) and GC (TS) 2.4 ± 0.1 (9.8 ± 0.5 mg/dL), *p* = 0.038
Fujita 2013 [56] Japan Cross-sectional	N = 73 (27 males, 46 females) **Age:** 20–45 years **Ethnicity:** Japanese **Inclusion:**: healthy Japanese volunteers **Exclusion:** N/A	*hKLK1* (promoter region, H allele: with nucleotide substitution -130(G)11) rs# not available	H allele frequency 24%	The common allele H polymorphism in Japanese may contribute to decreased reabsorptions of calcium and sodium in the kidney	Higher fractional urinary calcium excretion for subjects with H allele compared with subjects without H allele (0.9% ± 0.5 vs. 0.6% ± 0.5), *p* = 0.03, higher urinary calcium excretion in mg per mg creatinine for subjects with H allele compared with subjects without H allele (0.14 ± 0.09 vs. 0.09 ± 0.08), *p* = 0.03
Gianfagna 2013 [34] Belgium, Cyprus, Estonia, Germany, Hungary, Italy, Spain, Sweden Cross-sectional	N = 2267 (1188 males, 1079 females) **Age:** 6.2 ± 1.8 years **Ethnicity:** Caucasian **Inclusion:**: children participating in the large European multi-centre study IDEFICS with complete data on age, sex, parental questionnaire, height, weight, hip and waist circumferences, birthplace and language spoken at home as well as with provided saliva samples **Exclusion:** N/A	*NMU* rs9999653 (major/minor C/T)	CC 21.5%, CT 49.1%, TT 29.4%	The *NMU* gene plays a role through interaction with *ADRB2* gene in bone strength regulation (more evident in preschool girls)	*NMU* rs9999653 CC genotype was associated with lower serum calcium (*n* = 605), *p* = 0.01
Howles 2019 [35] UK, Japan Genome-wide association study	UK Biobank: 6536 cases, 388,508 controls; Biobank Japan: 5587 cases, 28,870 controls (from 4 population-based cohort studies); validation cohort: 440 patients **Age:** 40–69 years (UK Biobank) **Ethnicity:** Multi-ethnic **Inclusion:**: UK Biobank: history of nephrolithiasis; Biobank Japan: diagnosis of nephrolithiasis; validation cohort: kidney stone formers **Exclusion:** UK Biobank: disorder of calcium homeostasis, malabsorption, or other condition known to predispose to kidney stone disease; Biobank Japan: bladder stones	*CYP24A1* rs17216707 (T/C) *DGKD* rs838717(A/G)	*CYP24A1* (rs17216707) effect allele frequency T: UK Biobank 0.81, Biobank Japan 0.92; *DGKD* (rs838717) prevalence not described	In only nephrolithiasis patients, the *CYP24A1*-associated locus correlated with serum calcium concentration and a number of nephrolithiasis episodes while the *DGKD*-associated locus correlated with urinary calcium excretion	In validation cohort of kidney stone formers: significant higher serum calcium mmol/L (mg/dL) for *CYP24A1* (rs17216707) TT genotype 2.36 ± 0.01 (9.46 ± 0.04), *n* = 260 compared with TC genotype 2.32 ± 0.01 (9.30 ± 0.04), *n* = 109, but not CC genotype 2.34 ± 0.02 (9.38 ± 0.08), *n* = 15; significant lower 24 hr renal calcium excretion (mmol) in male patients carrying the *DGKD* (rs838717) AA genotype (4.54 ± 0.45, *n* = 33) compared with GG (7.27 ± 0.91, *n* = 25), but not AG genotype (5.45 ± 0.48, *n* = 57)
Hwang 2014 [36] Taiwan Case-control	N = 579 CKD patients (323 males, 256 females) **Age:** 6.2 ± 1.8 years **Ethnicity:** Taiwanese **Inclusion:**: >18 years of age with chronic kidney disease (CKD), detailed clinical history recorded as part of the CKD Care Program **Exclusion:** N/A Note: CKD patients divided into early-stage CKD (eGFR above 45 mL/min/1.73m^2^) and late-stage CKD (lower eGFR)	*ORAI1* rs12313273 (C/T)	Genotype frequency in *ORAI1* rs12313273: CC *n* = 50 (8.7%), CT *n* = 245 (42.5%), TT *n* = 281 (48.8%)	rs12313273 polymorphism was significantly associated with elevated serum calcium levels, which has been linked to increased risk of death in CKD patients	Significant association of *ORAI1* rs12313273 with serum calcium levels mmol/L (mg/dL) in CKD patients: CC genotype 2.33 ± 0.15 vs. CT 2.30 ± 0.14 vs. TT 2.27 ± 0.26 (9.32 ± 0.61 vs. 9.23 ± 0.57 vs. 9.08 ± 1.03), *p* = 0.0389; no differences between early and late stage
Kim 2018 [37] Korea Cross-sectional	N = 7815 (3629 males, 4186 females) **Age:** 51.7 ± 0.1 years **Ethnicity:** Korean **Inclusion:**: participants from the Ansung-Ansan cohort (community-based cohort) **Exclusion:** N/A	*GCKR* SNPs rs780093 (T/C); rs780094 (T/C); rs1260326 (T/C)	Genotype frequency for rs780093: TT *n* = 2247 (28.8%), TC *n* = 3895 (49.8%), CC *n* = 1673 (21.4%) rs780094: TT *n* = 2264 (29.0%), TC *n* = 3888 (49.8%), CC *n* = 1663 (21.3%) rs1260326: TT *n* = 2321 (29.7%), TC *n* = 3873 (49.6%), CC *n* = 1621 (20.7%)	Minor C allele carriers, particularly CC homozygotes, had lower serum calcium levels than TT homozygotes for all 3 SNPs. *GCKR* SNPs are associated with lipid profiles and glycaemic status in the Korean population (modified by basal circulating calcium levels in normal or high ranges)	Significantly decreased serum calcium level in participants with CC genotype (minor allele) compared with TT genotype but not TC for rs780093, rs780094, rs1260326
Koek 2010 [38] Netherlands Cross-sectional	N = 6146 (2369 males, 3777 females) **Age:** 55 years and older **Ethnicity:** Caucasian **Inclusion:**: participants from the Rotterdam study (large prospective population-based cohort study) **Exclusion:** N/A	*LPH* rs498823 (G/C, T-13910C, Thr > Cys)	Males: GG (TT) *n* = 1248 (48%), GC (TC) *n* = 1061 (41%), CC (CC) *n* = 281 (11%) females: GG (TT) *n* = 1816 (48%), GC (TC) *n* = 1577 (42%), CC (CC) *n* = 384 (10%)	Calcium intake and serum ionized serum calcium were significantly lower in C-homozygotes; no association between T-13910C polymorphism and VDR, bone mineral density or fractur	Dietary calcium intake (mg/day) lower in C-homozygotes (mean, SD): GG (TT) 1151 ± 352, GC (TC) 1120 ±3 69, CC (CC) 1058 ± 339, p trend = 3.0 × 10^−5^; lower ionized serum calcium mmol/L (mg/dL) in C-homozygotes (mean, SD): GG (TT) 1.29 ± 0.6 (5.17 ± 2.40), GC (TC) 1.29 ± 0.05 (5.17 ± 0.20), CC (CC) 1.28 ± 0.07 (5.13 ± 0.28), *p* = 0.02, but no difference in total serum calcium between genotypes
Masi 2007 [51] Italy Cross-sectional	N = 100 (25 males, 75 females) **Age:** 57 ± 8 years (range 47–76 years) **Ethnicity:** Caucasian **Inclusion:**: healthy Caucasian volunteers, no history of hypocalcemia **Exclusion:** N/A	*GNAS1* (nucleotide c.433-18T > C) rs# not available	TT *n* = 82 (82%), TC *n* = 15 (15%), CC *n* = 3 (3%)	A significant association with low serum calcium levels was found in healthy subjects carrying the novel *GNAS*1 T > C polymorphism	Lower serum calcium (mg/day) for individuals with C allele: TT 9.1 ± 0.9, TC 8.3 ± 0.87, CC 8.1 ± 0.25 (TT vs. TC *p* = 0.03; TT vs. CC *p* = 0.04)
Shakhssalim [47] 2014 Iran Case-control	N = 206 males (105 cases, 101 controls) **Age:** 30–55 years **Ethnicity:** N/A **Inclusion:**: cases: men with history of recurrent calcium urinary stones; controls: healthy volunteers without any personal or family history of urolithiasis **Exclusion:** cases and controls: histories of known metabolic, gastrointestinal, hepatic, renal and endocrinological diseases or any anatomic abnormality or obstruction in the urinary tract, taking any drugs which may affect urine composition	*CALCR* (3′UTR + 18C > T; rs# not reported) (9 SNPs studied, but only one associated with different calcium levels)	3′UTR + 18C > T: minor allele frequency in the population under study 7%; frequency in cases: wild type *n* = 73 (72.3%), heterozygotes *n* = 27 (26.7%), homozygotes *n* = 1 (1%); controls: wild type *n* = 101 (100%), hetero- and homozygotes *n* = 0 (0%)	Potential association of polymorphisms in the *CALCR* and the risk of kidney stone disease	3′UTR + 18C > T polymorphism in stone formers: significant difference in urine calcium concentration (mg/L 24 h) between wild type and C > T hetero- or homozygotes (mean ± SD): 117.13 ± 60.55 vs. 152.92 ± 72.18, *p* = 0.03), no difference in urine calcium mg/24 h or total serum calcium between wild type and hetero- or homozygotes

Abbreviations: ADRB2: beta-2-adrenergic receptor; AHSG: alpha 2-HS glycoprotein; CALCR: calcitonin receptor; CKD: chronic kidney disease; CLDN14: claudin-14; CYP24A1: cytochrome P450 family 24 subfamily A member 1; DGKD: diacylglycerol kinase delta; eGFR: estimated glomerular filtration rate; GCKR: glucokinase regulatory protein; GNAS1: guanine nucleotide binding protein alpha subunit; hKLK1: human renal kallikrein; LPH: lactase-phlorizin hydrolase; N/A: not available; NMU: neuromedin U; ORAI1: calcium release-activated calcium modulator 1; rs#: reference SNP ID; SD: standard deviation; SNP: single nucleotide polymorphism; UTR: untranslated region; VDR: vitamin D receptor; vs.: versus.

## Supplementary Materials

The following are available online at https://www.mdpi.com/article/10.3390/nu13082488/s1, Table S1: Search strategy in Medline and EMBASE.
