# Supplementary material for: Polymorphisms Contributing to Calcium Status: A Systematic Review
Source: Nutrients. 2021 Jul 21;13(8):2488. doi: 10.3390/nu13082488 (PMC8398213; doi:10.3390/nu13082488)
Supplement: Supplementary file 1 [file nutrients-13-02488-s001.zip › nutrients-1220162-supplementary.pdf]

Table S1. Search strategy in Medline and EMBASE on 7 August 2020.

#	Search History	Result
1	'calcium'/exp	305,528
2	calcium:ti	155,720
3	1 OR 2	387,129
4	(calcium NEAR/4 (plasma OR serum OR urin* OR bone OR skeletal)) OR (creatinine) OR (calcium NEAR/2 (metabolism OR status))	338,629
5	3 AND 4	54,239
6	(polymorphism$ OR genotyp* OR homozyg* OR heterozyg* OR genetic$ OR gene$ OR mutation$ OR snp$ OR hereditary OR inherited OR variant$ OR 'single nucleotide polymorphism$' OR gwas OR 'genome wide association' OR 'biomarker$' OR 'mendelian randomization'):ti	1,520,141
7	5 AND 6	1,676
8	('animal'/exp OR 'nonhuman'/exp OR 'animal cell'/exp OR 'animal cell culture'/exp OR ‘animal experiment’/exp OR ‘animal tissue’/exp OR ‘animal model’/exp) NOT (('animal'/exp OR 'nonhuman'/exp OR 'animal cell'/exp OR 'animal cell culture'/exp OR ‘animal experiment’/exp OR ‘animal tissue’/exp OR ‘animal model’/exp) AND 'human'/exp)	7,147,212
9	7 NOT 8	1,328
10	'case report'	2,627,358
11	9 NOT 10	1,005*

*Plus 7 additional hits form CENTRAL
